# Quantile regression of tobacco tax pass-through in the UK 2017–2021: how have manufacturers passed through tax changes for different tobacco products in small retailers? Analysis at the national level and by neighbourhood of deprivation

**DOI:** 10.1136/tc-2024-058958

**Published:** 2025-01-22

**Authors:** Luke B Wilson, Colin Angus, Alan Brennan, Duncan Gillespie, Niamh K Shortt, Helena Tunstall, Roberto Valiente, Jamie Pearce

**Affiliations:** 1 School of Medicine and Population Health, The University of Sheffield, Sheffield, UK; 2 SPECTRUM Consortium, , UK; 3 Centre for Research on Environment, Society and Health (CRESH), School of GeoSciences, University of Edinburgh, Edinburgh, UK

**Keywords:** Tobacco industry, Taxation, Economics, Price, Socioeconomic status

## Abstract

**ABSTRACT:**

**Background:**

The effectiveness of tax increases in reducing tobacco consumption relies on the tobacco retailers and producers passing on increases to consumers (tax pass-through). Previous UK research on supermarkets found heterogeneous levels of tax pass-through across the market segments and price distribution of tobacco products. This study uses data from small retailers across the UK to assess whether recent tax changes have been passed on to consumers and if this varies across the price distribution, between countries of the UK and by neighbourhood deprivation.

**Methods:**

We use panel data quantile regression analysis of tobacco sales in small retailers in the UK from March 2017 to December 2021 combined with UK tax rates and store-level index of multiple deprivation (IMD). We calculated the rate of tax pass-through for factory-made cigarettes (FM) and roll-your-own tobacco (RYO).

**Results:**

Following increases in the duty payable on tobacco, we find evidence of overshifting across the entire price distribution for FM and RYO. For England, Scotland and Wales, the rate of the overshift in tax increased with product price. For Scotland, we find that stores in the least deprived IMD pass-through taxes at a higher rate.

**Conclusions:**

Our evidence shows heterogeneous levels of tax pass-through by price, region and level of deprivation. The findings emphasise the importance of understanding the pricing strategies of the tobacco industry (TI) and how these vary across the UK to develop robust approaches to mitigate the pricing strategies of the TI.

**WHAT IS ALREADY KNOWN ON THIS TOPIC**

- Previous work in the UK which focused on large supermarkets has shown that the tobacco industry (TI) passed tax duty increases on to people who smoke at different magnitudes depending on the market and price segment of the product.
- The TI absorbs tax increases on the cheapest ‘sub-value’ and ‘value’ products, while simultaneously increasing the price, above the tax increase, of the relatively more expensive ‘premium’ products in order to maximise revenue.
- Less clear is how the rate of tax pass-through, following specific tax increases, varies across the price distribution of factory-made cigarettes (FM) and roll-your-own tobacco (RYO) as tobacco price segmentation overlaps by country of the UK and across level of neighbourhood deprivation.

**WHAT THIS STUDY ADDS**

- We create a counterfactual expected price taking into consideration specific duty increases and inflation in the UK and undertake quantile regression against transaction data to estimate tax pass-through of FM cigarettes and RYO across the price distribution of prices faced by people who smoke.
- The study fits panel data quantile regression on small retailer data, stratified by index of multiple deprivation to analyse tax pass-through by neighbourhood deprivation.
- The study extends previous work by applying panel data quantile regression methods to the monthly pricing data to produce estimates of undershifting or overshifting of tobacco tax changes at various price points across the entire distribution, covering the cheapest to most expensive products available.
- TI overshifts tax increases at a higher rate for the more expensive products relative to the cheapest products at the lower end of the price distribution. This is consistent for both FM and RYO.

**HOW THIS STUDY MIGHT AFFECT RESEARCH, PRACTICE OR POLICY**

- The variation found in this study of the extent of tax-related pricing changes in tobacco products across segments, regions and neighbourhoods demonstrates the need for robust approaches to mitigate the effects of price-related tobacco marketing in the UK.

## Introduction

 Tobacco consumption is higher in lower-income neighbourhoods, exacerbating economic inequalities.^1^,^3^ A substantial body of international evidence has documented the effectiveness of tobacco tax increases in reducing the consumption of tobacco and raising government revenue.^4^ However, the efficacy of tax increases in health-related outcomes relies heavily on the tobacco industry (TI) passing on such tax increases to retailers and consumers, also known as tax pass-through.^5^ Previous work has shown that, between 2000 and 2017 in the UK, the level of tax increases passed to people who smoke depended on the price and market segment of the product.^6^,^9^ This approach was operationalised by the TI absorbing tax increases on the cheapest products in the price distribution, or those that are marketed as ‘value’ or ‘sub-value’ versions (undershifting), while simultaneously increasing the price, above the tax increase, of the relatively more expensive products (overshifting). This approach to controlling tobacco prices by the TI aims to maximise revenue and maintain profits to mitigate the effects of declines in the number of tobacco consumers, while simultaneously lessening the impact of the price rise on the lowest-priced products. This helps to promote and maintain tobacco consumption in more price-sensitive consumers.^5^ Research conducted in the period prior to the introduction of standardised packaging in the UK (May 2016) found that the TI used a variety of strategies to maintain the price of lower-cost factory-made cigarettes (FM) and roll-your-own tobacco (RYO).^10^ These included price-marked packaging, introducing smaller pack sizes, as well as, absorbing the tax increase and accentuating the price gap between premium and ultra-low price products.^7 11 12^

UK analyses of recent tobacco tax increases and the introduction of standardised packaging has demonstrated that TI continued to adopt pricing strategies which maintain the price of the lowest cost tobacco products relative to the premium and more expensive products.^13 14^ This study found that the relative price of premium and value cigarette prices rose following the introduction of standardised packaging.^13^,^15^ Standardised packaging legislation requires all tobacco sold in the UK to be in packaging using the same typeface, be in plain boxes (Pantone 448 C), and have a minimum pack size of 20 FM cigarettes or 30 g of RYO tobacco. However, the study by Critchlow *et al* (2019) only included 20 of the leading tobacco products.^15^ Of which, more than half of the sample of cigarettes were in the ‘mid-price’ category which did see a fall in the relative price. Previous evidence from the UK found that ‘mid-price’ cigarettes make up 28% of all FM cigarettes sold annually in the UK.^7^

Neighbourhood differentials in tobacco prices are also important in understanding the substantial geographical variations in smoking rates.^16^ Previous research has found that consumers in low-income neighbourhoods tend to purchase cheaper products from small retailers.^17^ For example, compared with the least deprived quintile of neighbourhoods in Britain, the average price of FM and RYO in the most deprived quintile of neighbourhoods was around 5% lower than in the least deprived quintile, although this was in part accounted for by the different profile of products sold.^16^ In addition, small retailers in more deprived neighbourhoods stock the same brands at the same prices as less deprived neighbourhoods, however, the consumers in more deprived areas tend to buy more of the products at the cheaper end of the price distribution.^17^ Since tobacco consumers in lower-income neighbourhoods are more sensitive to price changes, it may be beneficial for the TI to ensure that tobacco tax increases have smaller effects on cheaper products sold in convenience stores in these areas. However, there is limited work examining spatial differences in tax pass-through and whether the TI adopts alternate pricing strategies in different types of communities. Understanding these geographical differences is important because it may provide new opportunities for innovative tobacco control approaches that combine price and place-based measures to reduce inequalities in tobacco consumption and tobacco-related health outcomes. A recent systematic review found that there are stark inequalities in tobacco retailer availability. The evidence showed consistent evidence of widespread neighbourhood inequities in tobacco retailer availability by socioeconomic status, ethnicity and race. They argued that place-based inequities may contribute to persistent inequities in exposure to tobacco marketing, tobacco use, as well as tobacco-related morbidity and mortality.^18^

This study uses electronic sales data from small retailers and convenience stores to investigate the extent to which tobacco tax changes are passed through to products sold at different prices per gram of tobacco throughout the UK. Our period of study is March 2017 to December 2021 which coincides with the introduction of standardised packaging and the Minimum Excise Duty (May 2017). Small retailers and convenience stores in this data represent 60% of all tobacco sales in the UK.^19^ First, we quantify the overall pattern of tobacco tax pass-through in small retailers. Second, we investigate whether tax pass-through varies by level of neighbourhood deprivation. We conduct this analysis separately for the four countries in the UK: England, Scotland, Wales and Northern Ireland. Our analysis applies panel data quantile regression analysis to estimate the geographic variation in tax pass-through. The results will show the extent to which neighbourhood deprivation is a factor in TI strategies for tax pass-through.

## Methods

### Data

Data were provided by The Retail Data Partnership (TRDP) from Electronic Point-of-Sale tills in convenience stores across the UK (https://shopmate.co.uk/).

The TRDP data is an item-level dataset in which each row is a sales record of all transactions (tobacco and non-tobacco). Data was provided for 4-weekly blocks (7–13 March, 7–13 June, 7–13 September and 7–13 December) each year from 2017 to 2021. We use these consistent weeks to account for any seasonality that may occur throughout the study period.

We focus on FM cigarettes and RYO. Each tobacco-related (Stock Keeping Unit) SKU contained information on the product name, specific European Article Number barcode, product type (FM or RYO), units (number of packages), pack size (number of sticks for FM products or grams for RYO), gross price (the price paid by the customer), as well as the census area in which the store was located (ie, Lower Layer Super Output Area comprising an average of 1700 population in England and Wales; data zones in Scotland with a mean population of 750 inhabitants; and Super Output Area in Northern Ireland with an average size of 2100 residents).

Neighbourhood level deprivation was indicated by the UK government measures of overall deprivation from the English Indices of Deprivation 2019 (EIoD 2019), Welsh Index of Multiple Deprivation 2019 (WIMD 2019), Scottish Index of Multiple Deprivation 2020 (SIMD 2020) and Northern Ireland Multiple Deprivation Measure 2017 (NIMDM 2017). Deprivation quintiles were defined separately for each nation, containing equal numbers of neighbourhoods, based on their deprivation ranking. As each country defines deprivation indices differently, the analysis was conducted differently for each country.

### Current tax structures in the UK

In the UK, tobacco tax structure is a reserved matter for the UK government at Westminster and consists of three types of tax. First, a specific tax, which are fixed amounts per 1000 FM cigarettes or per 1000 g of RYO tobacco. These lump-sum rates are different between FM and RYO. Second, a tobacco ad valorem tax, applied to FM, which is a proportion of the recommended retail price (16.5%), set in March 2011 and unaltered over the study period. Finally, an additional ad valorum sales tax, which is a general value added tax (VAT), applied to most goods and services on top of the retail price and has remained unchanged at 20% since January 2011. The level of tax paid on FM in the UK is further affected by the introduction of a minimum excise duty (MET) on FM cigarettes. This was introduced in May 2017. The MET is a separate fixed specific tax per stick which sets a price floor for the added total of two of the components (specific and tobacco ad valorem, but not VAT) charged to each product. If the total amount of specific and ad valorem tax payable on each product falls below the MET, then the tax payable is that of the MET rather than the sum of the specific and ad valorem tax elements.

### Estimating the effect of tax pass-through on product prices

To estimate the effects of tax passthrough, we constructed a counterfactual trajectory of tobacco prices over time. This assumes the TI keeps its profit margins constant for each product, so any tax increases have their intended impact on product sales prices without being affected by changes in industry profit margins. For every SKU of FM and RYO in our sample, we construct a monthly time series of the counterfactual expected price based on the ‘baseline’ actual price at the time point when the product is first observed in the TRDP data, and the expected impact of both inflation and specific duty changes in the following weeks of survey data available. Here, we assume that all specific duty increases are fully passed through to the consumer. Table 1 summarises all the specific duty changes in the UK between 2017 and 2021 in cash-term rates.

**Table 1:** Tobacco related tax changes in the UK during the period of analysis (cash-term rates)

Tax	March 2017	May 2017	November 2017	October 2018	March 2020	November 2020	October 2021
Panel A: specific excise duty for factory made and roll-your-own over time							
Factory made*	207.99	207.99	217.23	228.29	237.34	244.78	262.90
Minimum excise duty†		268.63	280.15	293.95	305.20	320.90	347.86
Roll your own‡	209.77	209.77	221.18	234.65	253.33	271.40	302.34
Panel B: change in specific excise duty from previous period (in £)							
Factory made		0	9.24	11.06	9.05	7.44	18.12
Roll your own		0	11.41	13.47	18.68	18.07	30.94
Panel C: percentage change in specific excise tax compared with previous period							
Factory made		0	+4.44	+5.09	+3.96	+3.13	+7.40
Roll your own		0	+5.44	+6.09	+7.96	+7.13	+11.40
Panel D: ad valorem and VAT tax rates over time							
Ad valorem tax§	16.5%	16.5%	16.5%	16.5%	16.5%	16.5%	16.5%
VAT	20%	20%	20%	20%	20%	20%	20%

* Specific tax £ per 1000 cigarettes.

† Minimum excise tax introduced for factory-made cigarettes: specific tax plus ad valorem tax (16.5%) of RRP.

‡ Specific tax £ per 1000 g.

§ Applied to factory made only.

A key variable of interest is the expected price per stick E[Price_it_]. This is the price per stick in pence for each SKU i assuming that there is full tax pass-through at the time of the specific duty change t. To calculate the expected price per stick, we first calculated the net revenue from each pack; this was done by removing all tobacco taxes for the relevant tobacco product applied at that point in time.

Once we calculated the ad valorem tax for FM, we converted all taxes payable for each SKU into per stick and calculated the evolution of expected price per stick for all tobacco products. Following existing literature, we assumed that RYO is 0.5 g per stick.^7 8 20^ The initial expected price per stick, E[Price_it_] takes the value of the observed price in the first time period it appears in the data. To construct the evolution of E[Price_it_] over time we removed the amount of the specific tax (Duty_it=1_), value-added tax (VAT) and $ad\;valorem\;tax_{it=1}$ for FM that would have been due at time t=1, this leaves only net revenue, see equation 1. Next, we checked whether the combined specific duty and ad valorem elements were higher or lower than the MET. If the MET was higher, we replaced the tax payable for these SKUs with the MET.


(1) $$Net\;revenue_{it=1}=\frac{\begin{array}{c} Retail\;price\;per\;stick_{it=1}\\ -Specific\;duty\;per\;stick_{it=1} \end{array}}{VAT\;{\%}_{t=1}}\begin{array}{c} -Ad\;valorem\;tax\\ per\;stick_{it=1} \end{array}$$

Net revenue is the money the TI retains from its sales once all taxes and VAT have been paid. This forms a ‘baseline’ price, which is then inflated to real terms using the Retail Price Index (RPI_t_). We use RPI as opposed to other measures of inflation as this is used to set the path for the Tobacco Duty Escalator in the UK as well as other products such as alcohol. RPI is a measure of inflation published monthly by the Office for National Statistics. It measures the change in the cost of a representative sample of retail goods and services. The UK Treasury uses RPI for various index linked tax rises. Our inflated baseline price is then updated over the time frame of the study to reflect the incremental change in specific tax in each following time period. Similarly, if a product’s price increases exactly in line with specific tax, VAT and inflation, then its expected price is equal to the observed price. Our estimated equation to get expected price becomes as follows:


(2) $$E\;[Price_{it}]=\begin{array}{l} \left((Net\;revenue_{it=1}\times RPI_{t}\right)\\ +Specific\;duty\;per\;stick_{it})\times (VAT\;{\%}_{t})\\ +Ad\;valorem\;tax\;per\;stick_{it}) \end{array}$$

### Panel data quantile regression strategy to estimate tax pass-through across the price range

Similar to analysis examining tax pass-through in the alcohol industry,^21 22^ we exploit the panel nature of the price data and adopt a quantile regression approach.^23 24^ Rather than focusing only on the predicted mean of the dependent variable, as in classical linear regression, quantile regression focuses on quantiles which refer to defined points in the price distribution. For example, the 0.50 quantile is the median and 0.05 is the 5th percentile (the cheaper end) of the distribution. This allows for the flexibility for modelling the entire distribution of observed prices as the dependent variable. This methodology provides a framework for investigating differential tax pass-through for price points across the entire price distribution. Covariates representing socioeconomic deprivation can then be introduced to investigate differences in the patterns of tax pass-through. We fit separate models for each country.

The basic version of our model is as follows:


(3) $$Observed\;price_{it}=\begin{array}{l} \beta_{0}+\beta_{1,\theta }Expected\;price_{it}\\ +\varepsilon_{it,\theta } \end{array}$$

Where Observed price_it_ is the observed price per stick of SKU i at time t in the TRDP data and Expected price_it_ is the price per stick calculated counterfactual price assuming a full tax pass-through.

We consider 11 quantiles (0.05, 0.15, 0.25, 0.35, 0.45, 0.5, 0.55, 0.65, 0.75, 0.85, 0.95) which includes the median θ=0.50 to try and fully capture the price distribution of tobacco. We run the model for FM and RYO separately.

If tax changes are fully passed onto people who smoke across the price distribution, then, for all quantiles, the estimated *β*_1_ coefficient of a given SKU should equal 1. If *β*_1_ is less than 1, this is an example of undershifting, and the producer is losing some revenue and bears some of the burden of the tax change. If *β*_1_ is greater than 1, this represents overshifting and the person smoking is paying more than the 100% tax pass-through expected price given the tax change, and the TI or retailer is gaining additional revenue.

## Results

**Figure 1:**
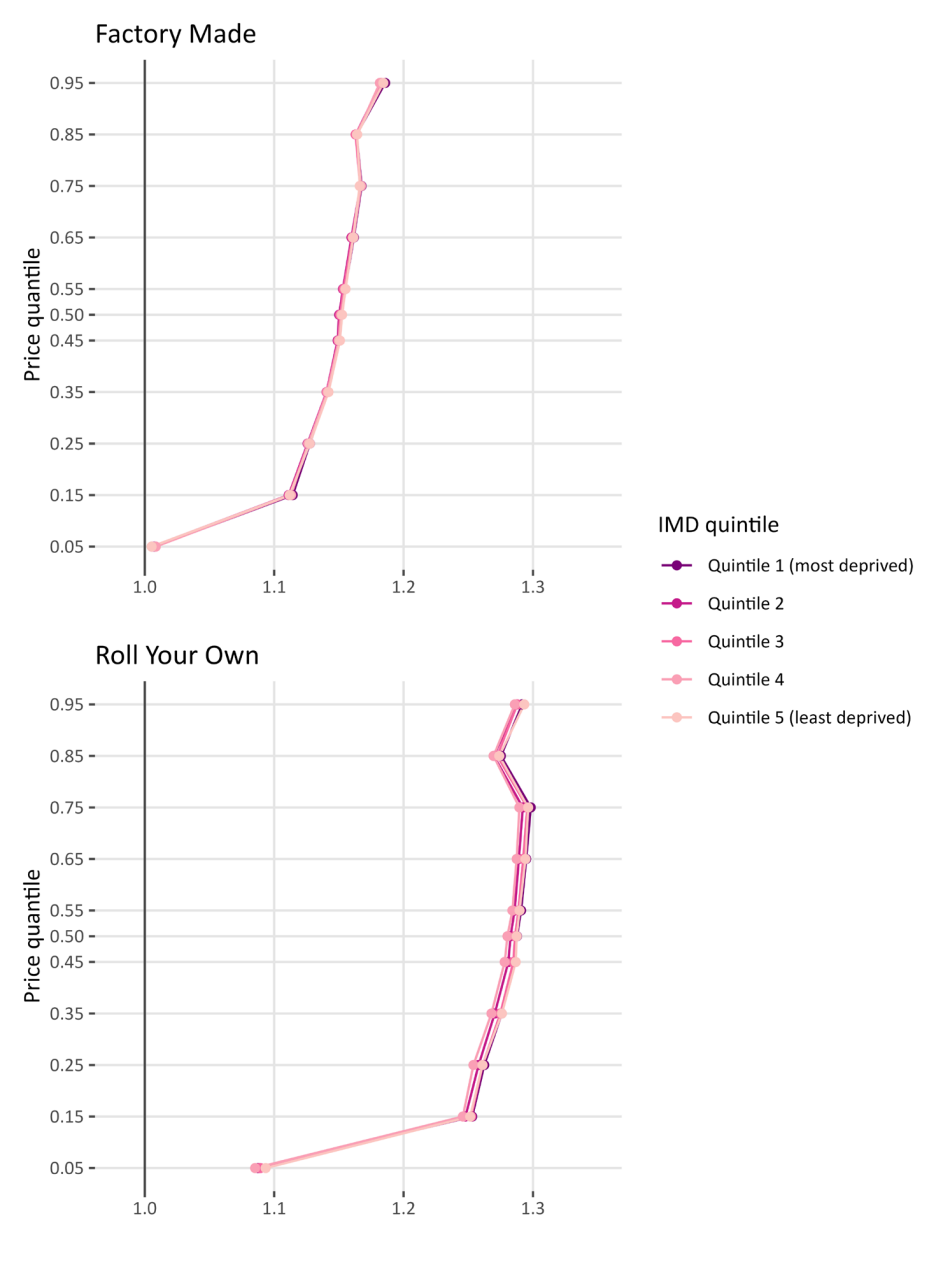
Tobacco tax pass-through for England for factory-made cigarettes and roll-your-own tobacco by index of multiple deprivation (IMD).

Figure 1 displays the tobacco-specific tax pass-through estimates for FM and RYO in England, stratified by neighbourhood deprivation of the store (IMD 1 is the most deprived and IMD 5 is the least deprived). For FM we find that at the lowest end of the price distribution (θ≤0.05), tax pass-through is close to 100% tax pass-through and this finding is consistent across the deprivation quintiles (IMD 1 (most deprived) 1.01 (1.01, 1.01) and IMD 5 (least deprived) 1.01 (1.01, 1.01)). On the other hand, at the 5% cheapest set of products (θ≤0.05) for RYO, there is an overshift for the cheapest products IMD 1 (most deprived) 1.09 (1.09, 1.09) and IMD 5 (least deprived) 1.09 (1.09, 1.09). See online supplemental materials for numerical values for all figures presented.

For the remaining quantiles, there is a clear overshifting of tax onto consumers. However, the overshift is smaller for products at the lower end of the price spectrum. At θ=0.25, tax pass-through for all IMD quintiles is 1.13 (1.13 1.13) for FM and 1.26 (1.25 1.26) for RYO while at θ=0.75 overshifting increases to 1.17 (1.17 1.17) for FM and is 1.29 (1.29 1.30) for RYO. In England, for both FM and RYO we find little evidence to suggest that tax pass-through varies by IMD.

**Figure 2:**
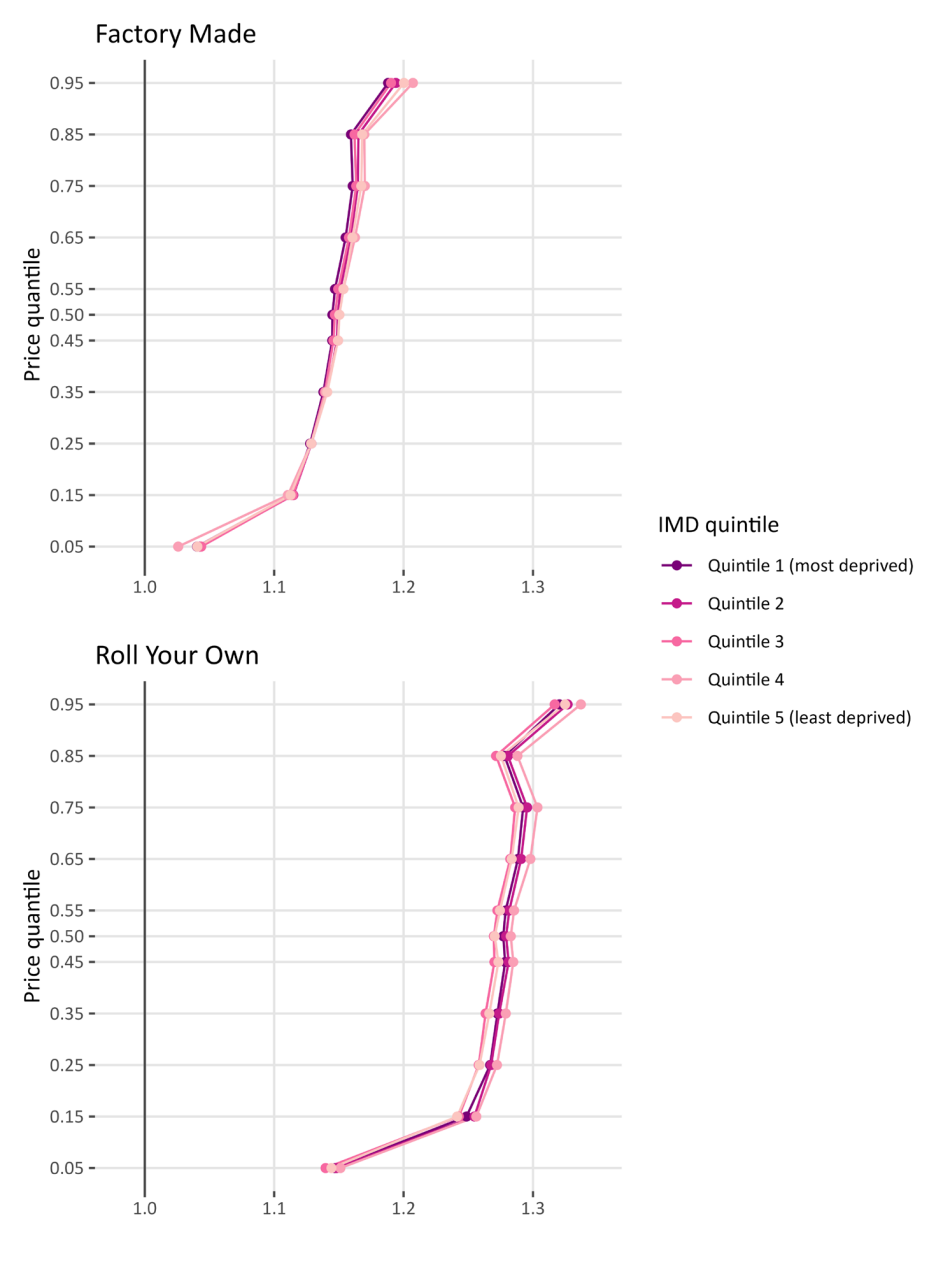
Tobacco tax pass-through for Scotland for factory-made cigarettes and roll-your-own tobacco by index of multiple deprivation (IMD).

Similar to England, in Scotland we find that at the lowest quintile (θ≤0.05), tobacco taxes are overshifted however at a lower rate than the rest of the distribution (figure 2). This is again consistent for FM (1.04 (1.04 1.04)) and RYO (1.15 (1.14 1.15)). As we move up the price distribution the overshifting increases. At θ=0.25 tax pass-through increase to 1.13 (1.13 1.13) for FM and 1.27 (1.26 1.27) for RYO. At the top quantile θ=0.95 the tax overshift is at its peak for FM and RYO.

As the price increases, there is evidence to suggest a difference in tax pass-through depending on the IMD, although the magnitude of the between-IMD differences in pass-through is much smaller than the variation across the price distribution. At θ=0.75 the stores in the most deprived IMD (IMD 1) have a pass-through rate of 1.16 (1.16 1.16) while those in the least deprived (IMD 5) have a higher pass-through rate 1.17 (1.17 1.17) (p<0.01). This pattern is also visible in the RYO panel with IMD 4 and IMD 5 having higher tax pass-through than the other IMD quintiles.

**Figure 3:**
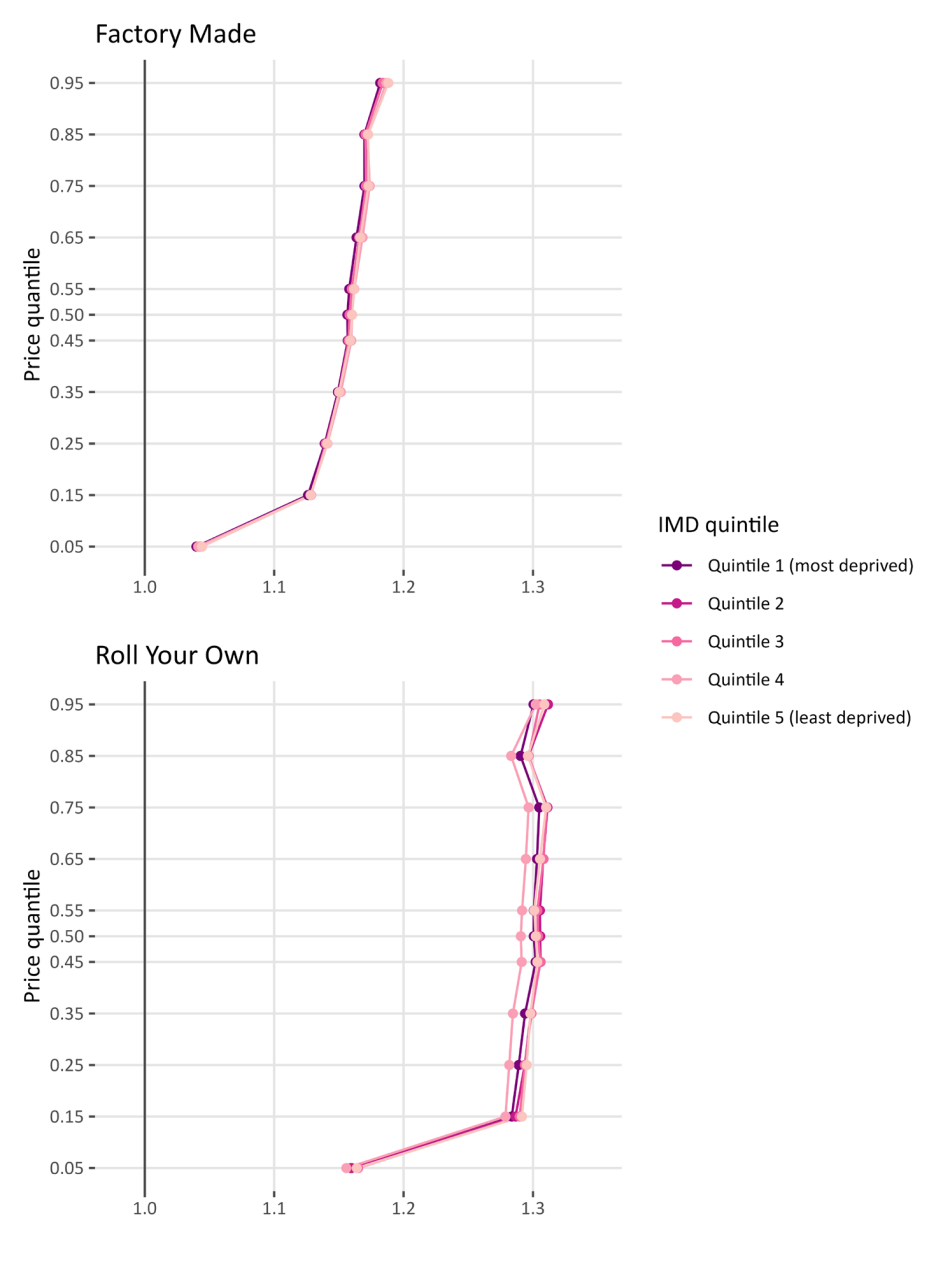
Tobacco tax pass-through for Wales for factory-made cigarettes and roll-your-own tobacco by index of multiple deprivation (IMD).

The shape of the tax pass-through curve in Wales is similar to that of the results presented for England and Scotland (figure 3). Again, we find evidence to suggest that tax pass-through is overshifted throughout but is lowest for the cheapest products available in Wales. Overshifting increases in magnitude the higher the prices for FM, however this pattern is less obvious for RYO. With respect to differences in pass-through by IMD, we find little statistical difference between pass-through rates for FM and no clear pattern for RYO.

**Figure 4:**
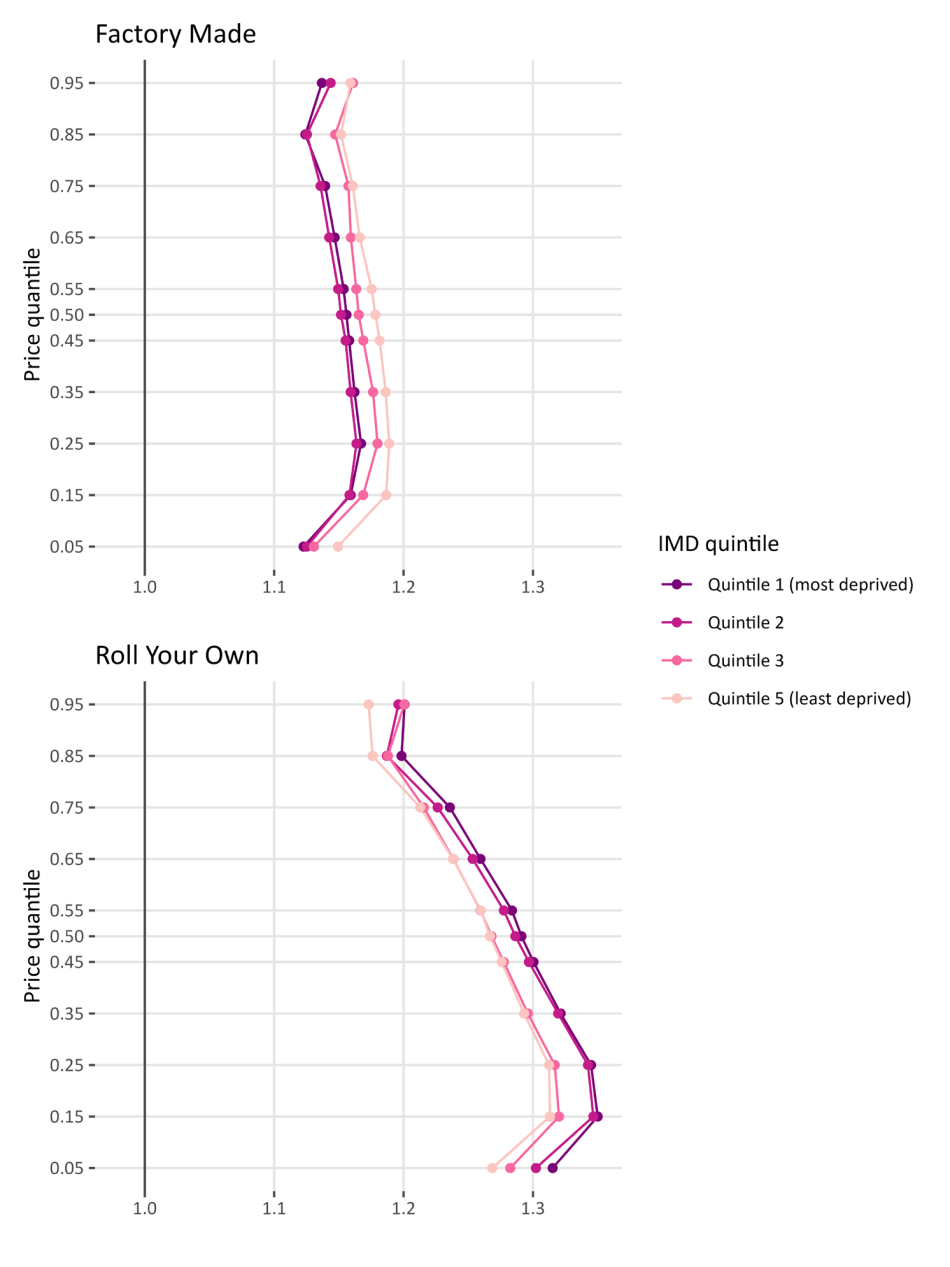
Tobacco tax pass-through for Northern Ireland for factory-made cigarettes and roll-your-own tobacco by index of multiple deprivation (IMD).

In Northern Ireland for FM, similar to the results for the other three UK nations (figure 4), we find that at θ≤0.05 the tax overshift is at its lowest for all IMD quintiles. However, contrary to the previous results we find that the products within the lowest 50% of the price distribution (excluding the lowest quintile) are in fact overshifted at a higher rate than the 50% more expensive products. This is consistent for both FM and RYO. We find that for RYO the lower priced products are overshifted at a higher rate than the more expensive products. In addition, we also find that the stores in the more deprived areas of Northern Ireland pass-through taxes at a higher rate than stores in the least deprived areas.

## Discussion

We used quantile regression for panel data to estimate the rate of tax pass-through in the market for tobacco in small retailers and convenience stores in the UK between 2017 and 2021. In small retailers, we find that the TI predominantly overshifts tax in the form of higher-than-expected prices, a finding that was consistent in all four countries of the UK. This is consistent with evidence presented in a recent systematic review which found that in 15 out of 22 studies the TI persistently raised tobacco prices by overshifting tax increases on tobacco products at the higher end of the market but undershifted on products in the lowest segments to keep their prices cheap.^5^ Furthermore, we find that the size of the overshift is larger for the more expensive FM and RYO products at the higher end of the price distribution. These findings are similar with recent evidence from the USA which found that cigarette excise taxes were fully shifted or overshifted to prices, with the tax pass-through rate higher for higher-priced cigarettes.^25^ In order to understand the potential implications of tax pass-through for health equity, we also stratified the analysis by a measure of neighbourhood deprivation. While we found little evidence that the IMD gradient in pass-through differed between England and Wales, in Scotland tobacco prices at the higher end of the price distribution are overshifted at stores in more affluent areas (IMD 4 and IMD 5) relative to stores in more deprived areas (IMD 1 and IMD 2). This suggests that tax pass-through is generally consistent across different areas. However, in some cases, small retailers in more affluent areas have been increasing profits more on higher-priced products. We find that the between-IMD variation is relatively small when compared with the between-percentile variation of prices. Even in areas of high deprivation, the people who smoke buying expensive cigarettes are subsidising those buying cheaper cigarettes, to a much greater extent than people who smoke in affluent areas are subsidising those buying cigarettes at similar price points in more deprived areas. We find conflicting results for Northern Ireland, with our analysis showing greater overshifting for cheaper products. It is unclear if this reflects greater uncertainty in the data, due to the relatively smaller size of the Northern Irish market, or if it is a genuine effect driven by different market conditions in Northern Ireland. Further research to explore the robustness of and, if appropriate; possible explanations for, this finding would be welcome.

Following similar analysis published for large retailers for the UK as a whole,^6^ we find evidence that the TI overshift tax changes onto consumers. The level of overshift is lower for the cheapest products, however the magnitude of the overshift increases relative to price. Evidence for Scotland found the purchase price of FM and RYO was lower in more income-deprived neighbourhoods, and that the lower prices primarily reflect greater sales of cheap brands in these areas, rather than retailers reducing the prices of individual brands. However, we find little evidence of IMD differences in tax pass-through. This result is consistent across all four nations of the UK. This result is important when considering policy changes and predicting the potential policy impact. Local variation in policy impacts is primarily driven by local-level price distributions with little additional influence on local-level tax pass-through estimates.

Increasing taxation on unhealthy commodities including tobacco products is widely regarded as a highly effective strategy to reduce consumption,^4^ improve population health and address inequalities in tobacco-related outcomes.^26^ Measures to raise taxation on tobacco also form a key component of tobacco control approaches in many countries including the smoke-free ambitions of the UK nations. While the potential of tobacco taxation to improve population health is clear, the public health benefits of rises in taxation are contingent on the TI passing on price increases to the consumer and can be undermined by tactics such as absorbing tax increases for the lowest-cost products. It is therefore important to assess the pricing strategies of the TI, particularly following the introduction of new tobacco control measures affecting product pricing.

The main strength of our analysis is our use of quantile regression for panel data to estimate the rate of tax pass-through in the market for tobacco in small retailers across different geographies by income deprivation context. This is the first study to break the market into consistent, exogenously defined, quantiles and estimate tax pass-through for four nations in the UK and by deprivation. This study is one the first to provide detailed insight into how the TI may alter pricing strategies of their products based on store location.

While we provide new evidence regarding the extent of tax pass-through by the TI, our work is not without limitations. The focus of this study is FM cigarettes and RYO. There is a growing proportion of the UK population who are using electronic cigarettes (e-cigarettes). The most recent estimates published in 2023 show that, 9.1% of adults, 7.2% of 11 to 17-year-olds regularly use e-cigarettes.^27 28^ Due to data constraints we are unable to control for e-cigarette use in our analysis. However, evidence from the USA found that the effect of e-cigarette evolution on cigarette tax pass-through rates was not significant after they controlled for state-specific year trends.^25^ Due to commercial sensitivity, we are unable to obtain the exact ad valorem rate payable or RRP suggested by the TI for each SKU in the TRDP data. Instead, we use similar methods from the previous literature as well as cross-checking against the cost price paid by the retailer for each SKU as well as conversations with His Majesty’s Revenue and Customs to calculate the ad valorem tax. In addition, the sample of stores from the TRDP is an opportunity sample and may not be representative of all convenience stores. Each nation defines its deprivation indices differently, therefore comparison of IMD gradients between UK nations is challenging, due to the differences in the definition of IMD, and the underlying indicators used to generate IMD rankings within each country. However, previous research seeking to produce a harmonised IMD measure to allow for such comparisons has found that there was little divergence for Scotland between the original SIMD rankings and those under the harmonised measure.^29^

Further, most current estimates of price elasticity for tobacco examine average mean changes in sales or purchasing. Future research could focus on differential price elasticities at different price points across the price distribution or across product segments. Our research illustrates that the TI passes on tax increases heterogeneously across the price distribution; an extension to this can be to examine how people who smoke respond to the price increases they face.

## Conclusion

Our results indicate that tax increases lead to increases in the price of FM cigarettes and RYO across the price distribution. We also find evidence to suggest that the TI pass-through taxes at different magnitudes depending on price by overshifting taxes at a lower rate for cheaper products in England, Scotland and Wales. These findings have important policy implications, particularly in jurisdictions such as the nations of the UK which are currently developing new tobacco control strategies aimed at creating ‘smoke-free generations’ within 10–15 years. Price is an important policy tool to prevent uptake of smoking and aid cessation, therefore it is important to understand pricing strategies across all nations of the UK and to develop robust approaches to mitigate the pricing tactics of the TI.

## Supplementary material

10.1136/tc-2024-058958 online supplemental file 1

## Data Availability

Data may be obtained from a third party and are not publicly available.
