# Supplementary material for: Quantile regression of tobacco tax pass-through in the UK 2017–2021: how have manufacturers passed through tax changes for different tobacco products in small retailers? Analysis at the national level and by neighbourhood of deprivation
Source: Tob Control. 2025 Jan 22;35(3):e058958. doi: 10.1136/tc-2024-058958 (PMC13217080; doi:10.1136/tc-2024-058958)
Supplement: online supplemental file 1 [file tc-35-3-s001.pdf]

Quantile regression of tobacco tax pass-through in the UK 2017- 2021: How have manufacturers passed through tax changes for different tobacco products in small retailers? Analysis at the national level and by neighbourhood of deprivation.

Luke B. Wilson[1]

Colin Angus[1,3]

Alan Brennan[1,3]

Duncan Gillespie[1,3]

Niamh Shortt[2,3]

Helena Tunstall[2,3]

Roberto Valiente[2,3]

Jamie Pearce[2,3]

[1] Sheffield Centre for Health and Related Research (SCHARR), University of Sheffield, Sheffield, S1 4DA, UK

[2] Centre for Research on Environment, Society and Health (CRESH), School of GeoSciences, University of Edinburgh, Edinburgh EH8 9XP, UK.

[3] SPECTRUM Consortium, UK.

[*] Corresponding author. Address: Sheffield Centre for Health and Related Research, University of Sheffield Email: l.b.wilson@sheffield.ac.uk

Corresponding author telephone: 0114 222 0815

Corresponding author ORCID: 0000-0001-5769-5729

Supplementary Materials

Table A1: Quantiles of prices paid per stick* for factory made and roll your own tobacco for England

	Factory Made					Roll Your Own				
	1 (Most Deprived)**	2	3	4	5 (Least Deprived)	1 (Most Deprived)	2	3	4	5 (Least Deprived)
θ≤0.05	0.334	0.334	0.341	0.334	0.341	0.190	0.191	0.192	0.192	0.190
0.05<θ≤0.15	0.357	0.360	0.361	0.361	0.364	0.201	0.203	0.203	0.201	0.202
0.15<θ≤0.25	0.376	0.378	0.378	0.381	0.383	0.209	0.207	0.209	0.208	0.206
0.25<θ≤0.35	0.392	0.395	0.397	0.401	0.405	0.213	0.214	0.213	0.212	0.213
0.35<θ≤0.45	0.414	0.418	0.420	0.426	0.433	0.224	0.224	0.226	0.224	0.226
0.45<θ≤0.50	0.425	0.429	0.435	0.442	0.446	0.231	0.227	0.229	0.227	0.231
0.50<θ≤0.55	0.442	0.442	0.449	0.453	0.456	0.237	0.233	0.232	0.231	0.236
0.55<θ≤0.65	0.468	0.469	0.475	0.479	0.490	0.246	0.245	0.244	0.247	0.244
0.65<θ≤0.75	0.499	0.505	0.509	0.511	0.513	0.254	0.260	0.253	0.254	0.252
0.75<θ≤0.85	0.540	0.549	0.553	0.553	0.560	0.267	0.267	0.267	0.268	0.267
0.85<θ≤0.95	0.616	0.625	0.625	0.628	0.633	0.275	0.276	0.277	0.278	0.276
0.95>θ	0.707	0.770	0.900	0.709	0.984	0.287	0.288	0.289	0.291	0.288

Notes: *Price per stick refers to the upper bound of each quantile band (θ). ** Refers to IMD Quintiles for that particular country, IMD 1 refers to the most deprived IMD 5 refers to the least deprived.

Table A2: Quantiles of prices paid per stick* for factory made and roll your own tobacco for Scotland

	Factory Made					Roll Your Own				
	1 (Most Deprived)**	2	3	4	5 (Least Deprived)	1 (Most Deprived)	2	3	4	5 (Least Deprived)
θ≤0.05	0.340	0.342	0.350	0.343	0.352	0.180	0.179	0.184	0.183	0.186
0.05<θ≤0.15	0.361	0.370	0.379	0.375	0.383	0.193	0.190	0.193	0.192	0.193
0.15<θ≤0.25	0.385	0.392	0.405	0.400	0.416	0.201	0.202	0.203	0.205	0.205
0.25<θ≤0.35	0.410	0.417	0.429	0.432	0.440	0.207	0.206	0.209	0.212	0.208
0.35<θ≤0.45	0.430	0.442	0.450	0.452	0.465	0.216	0.212	0.222	0.217	0.216
0.45<θ≤0.50	0.442	0.450	0.463	0.466	0.473	0.225	0.224	0.226	0.224	0.222
0.50<θ≤0.55	0.450	0.461	0.475	0.477	0.483	0.231	0.227	0.229	0.233	0.223
0.55<θ≤0.65	0.477	0.487	0.498	0.500	0.502	0.240	0.235	0.238	0.242	0.238
0.65<θ≤0.75	0.502	0.507	0.513	0.521	0.521	0.246	0.245	0.247	0.249	0.247
0.75<θ≤0.85	0.539	0.549	0.555	0.563	0.558	0.265	0.264	0.267	0.267	0.266
0.85<θ≤0.95	0.605	0.603	0.610	0.646	0.625	0.279	0.276	0.277	0.276	0.279
0.95>θ	0.747	0.681	0.722	0.794	0.712	0.283	0.283	0.285	0.287	0.296

Notes: *Price per stick refers to the upper bound of each quantile band (θ). ** Refers to IMD Quintiles for that particular country, IMD 1 refers to the most deprived IMD 5 refers to the least deprived.

Table A3: Quantiles of prices paid per stick* for factory made and roll your own tobacco for Wales

	Factory Made					Roll Your Own				
	1 (Most Deprived)**	2	3	4	5 (Least Deprived)	1 (Most Deprived)	2	3	4	5 (Least Deprived)
θ≤0.05	0.342	0.342	0.342	0.349	0.352	0.187	0.185	0.187	0.180	0.178
0.05<θ≤0.15	0.368	0.367	0.367	0.380	0.381	0.201	0.197	0.199	0.195	0.192
0.15<θ≤0.25	0.384	0.387	0.387	0.410	0.411	0.207	0.203	0.206	0.206	0.204
0.25<θ≤0.35	0.410	0.412	0.413	0.433	0.437	0.214	0.211	0.210	0.211	0.209
0.35<θ≤0.45	0.436	0.438	0.438	0.451	0.453	0.226	0.219	0.222	0.226	0.217
0.45<θ≤0.50	0.445	0.445	0.447	0.468	0.471	0.229	0.225	0.228	0.229	0.219
0.50<θ≤0.55	0.454	0.454	0.457	0.479	0.477	0.234	0.230	0.231	0.235	0.224
0.55<θ≤0.65	0.485	0.478	0.484	0.497	0.500	0.240	0.240	0.241	0.239	0.232
0.65<θ≤0.75	0.506	0.503	0.507	0.516	0.518	0.250	0.242	0.252	0.248	0.244
0.75<θ≤0.85	0.539	0.534	0.549	0.556	0.554	0.268	0.266	0.266	0.264	0.263
0.85<θ≤0.95	0.604	0.581	0.606	0.629	0.609	0.280	0.276	0.278	0.272	0.280
0.95>θ	0.694	0.688	0.683	0.689	0.745	0.296	0.289	0.282	0.281	0.286

Notes: *Price per stick refers to the upper bound of each quantile band (θ). ** Refers to IMD Quintiles for that particular country, IMD 1 refers to the most deprived IMD 5 refers to the least deprived.

Table A4: Quantiles of prices paid per stick* for factory made and roll your own tobacco for Northern Ireland

	Factory Made					Roll Your Own				
	1 (Most Deprived)**	2	3	4	5 (Least Deprived)	1 (Most Deprived)	2	3	4	5 (Least Deprived)
θ≤0.05	0.374	0.364	0.424		0.432	0.192	0.182	0.192		0.180
0.05<θ≤0.15	0.415	0.409	0.445		0.454	0.196	0.190	0.200		0.207
0.15<θ≤0.25	0.443	0.441	0.462		0.473	0.201	0.196	0.208		0.211
0.25<θ≤0.35	0.467	0.456	0.478		0.498	0.203	0.203	0.214		0.220
0.35<θ≤0.45	0.481	0.475	0.508		0.520	0.210	0.210	0.233		0.226
0.45<θ≤0.50	0.488	0.485	0.515		0.528	0.213	0.217	0.233		0.226
0.50<θ≤0.55	0.498	0.493	0.520		0.530	0.223	0.222	0.233		0.233
0.55<θ≤0.65	0.509	0.505	0.533		0.539	0.225	0.228	0.248		0.243
0.65<θ≤0.75	0.530	0.521	0.560		0.563	0.236	0.239	0.252		0.251
0.75<θ≤0.85	0.559	0.547	0.589		0.594	0.244	0.243	0.258		0.266
0.85<θ≤0.95	0.615	0.602	0.645		0.642	0.259	0.259	0.280		0.288
0.95>θ	0.688	0.682	0.710		0.702	0.274	0.271	0.304		0.293

Notes: *Price per stick refers to the upper bound of each quantile band (θ). ** Refers to IMD Quintiles for that particular country, IMD 1 refers to the most deprived IMD 5 refers to the least deprived.
